# Number of metastasis-positive lymph node stations is a simple and reliable prognostic factor following surgery in patients with esophageal cancer

**DOI:** 10.3892/etm.2012.705

**Published:** 2012-09-13

**Authors:** SHINSUKE TAKENO, SHIN-ICHI YAMASHITA, SATOSHI YAMAMOTO, YOSHIAKI TAKAHASHI, TOSHIHIKO MOROGA, KATSUNOBU KAWAHARA, TOYOO SHIROSHITA, IPPEI YAMANA, KENJI MAKI, YUICHI YAMASHITA

**Affiliations:** 1 Department of Gastroenterological Surgery, Fukuoka University Faculty of Medicine, Fukuoka City, Fukuoka;; 2 Department of Surgery II, Oita University Faculty of Medicine, Oita, Japan

**Keywords:** esophageal cancer, lymph node metastasis, lymph node station, TNM classification, Japanese classification

## Abstract

The aim of this study was to evaluate the utility of lymph node metastasis classification based on the number of positive stations in patients undergoing surgical management of esophageal cancer. Of 257 patients who underwent curative esophagectomy, 126 patients with lymph node involvement underwent assessment of nodal metastasis mode according to the 7th edition of the TNM classification (UICC), and the Japanese Guidelines for the Clinical and Pathological Studies on Carcinoma of the Esophagus. Lymph node metastasis mode was divided into single station (S) and multi-station (M) groups. The S group was subclassified into single-node-single-station (SS) or multi-node-single-station (MS), and the M group was subclassified into multi-station in pN1 (2 metastasis positive nodes; MM-pN1) or multi-station in pN2 or 3 (MM-pN2,3) by TNM classification, multi-station-single-area (MMS) or multi-station-multi-areas (MMM). The correlation between prognosis and lymph node metastasis mode was assessed. A total of 47 patients were classified as S (MS, n=11; SS, n=36), and 79 patients were classified as M (MM-pN1, n=12; MM-pN2,3, n=67; MMM, n=55; MMS, n=24). Prognosis was poorer among the M- than in the S-classified patients (p=0.0035), whereas prognosis was not significantly different between the subgroups. In conclusion, lymph node metastasis classification based on the number of metastasis-positive stations is a useful predictor of prognosis in patients undergoing surgical management of esophageal cancer. This system relies on a simple classification method that combines the Japanese classification based on lymphatic spread and the TNM classification based on the number of positive lymph nodes.

## Introduction

Esophageal cancer ranks among the ten most common cancers in the world, and lymph node metastasis is a critical determinant of poor prognosis for this cancer (1,2). Lymph node classification is based on the number of nodes with metastatic foci within the most recent (7th) edition of the tumor, node, metastasis (TNM) system of classification defined by the International Union Against Cancer (UICC) (3). This reflects a distinct prognostic difference from the 6th edition of these guidelines, in which nodal metastasis was graded as either present or absent. By contrast, the lymph node classification endorsed by the Japanese Society for Esophageal Disease is based on anatomical lymphatic spread to lymph node stations and is also useful for the assessment of prognosis (4). However, to date, there is no consensus as to which nodal classification system is the most useful for assessment of prognosis, although several other nodal classification have been proposed.

The aim of this study was to examine the utility and feasibility of a novel metastatic node classification system that combines the intensity of node metastasis (represented by the TNM classification system) and anatomical lymphatic spread (represented by the Japanese classification system) for the assessment of prognosis of patients undergoing surgical management for esophageal cancer.

## Patients and methods

### Patients

Data were obtained from 257 patients (224 males and 33 females; mean age, 64.0 years) who underwent transthoracic esophagectomy via the right transthoracic route for esophageal cancer without preoperative chemotherapy or radiotherapy between January 1991 and December 2008. Data were collected and analyzed retrospectively, and all patients employed in the analysis were followed until death or until December 2010 (i.e., at least 2 years after surgery).

Clinicopathological characteristics including tumor invasion, node metastasis and stage were based on the TNM classification, 7th edition, by the International Union Against Cancer, and on the Japanese Guidelines for the Clinical and Pathologic Studies on Carcinoma of the Esophagus. Lymph node station spread was determined according to the Japanese classification system (3,4).

### Classification of lymph node status

The mode of lymph node metastasis was divided into two groups: single-station (S) and multi-station (M). In addition, the S group was subclassified into a single-node-single-station (SS) group, in which lymph node metastasis was detected in only one node, and a multi-node-single-station (MS) group, in which lymph node metastasis was detected in two or more nodes within a single lymph node station. Furthermore, the M group was also subclassified into a multi-station in pN1 (two metastasis-positive nodes) by TNM classification (MM-pN1) group, a multi-station in pN2 or 3 in TNM classification (MM-pN2,3) group, a multi-station-single-area (MMS) group, in which the metastasis-positive lymph node station was localized to the cervical, thoracic or abdominal area, and a multi-station-multi-area (MMM) group, in which metastasis-positive nodes were present in two or more of these areas.

### Statistical analysis

The correlation between prognosis and lymph node metastasis mode was assessed. The Kaplan-Meier method by Wilcoxon test was used to assess prognosis after surgery. A p-value <0.05 was considered to indicate statistical significance in each analysis.

## Results

**Figure 1:**
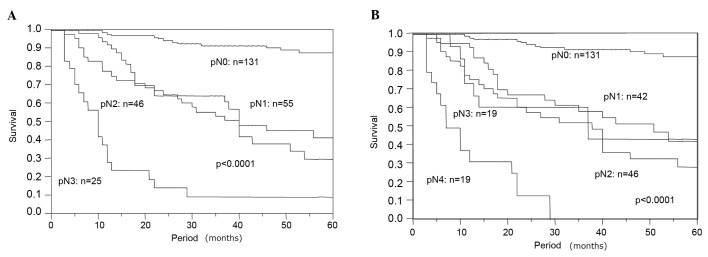
Survival based on the lymph node metastasis category of (A) TNM and (B) Japanese classifications.

Patient characteristics are shown in Table I. Of the 257 patients, 131 (51.0%) had no lymph node metastasis. Of the 126 patients with lymph node metastasis, 55 patients (43.7%) were classified as pN1, 46 (36.5%) were classified as pN2, and 25 (19.8%) were classified as pN3 by pN category of the TNM classification based on the number of metastasis-positive lymph nodes. By contrast, 42 patients (33.3%) were classified as pN1, 46 (36.5%) were classified as pN2, 19 (15.1%) were classified as pN3 and 19 (15.1%) were classified as pN4 by the Japanese classification system based on lymphatic spread to lymph node station. Disease-specific survivals according to TNM and Japanese classifications are shown in Fig. 1A and B, and both classifications revealed significant prognostic differences between pN categories (p<0.0001).

Of the 126 patients who were node metastasis-positive, 47 (37.3%) were classified as S group, and 79 patients (62.7%) were classified as M group. Among the S-group patients, 11 (23.4%) were classified as the MS group, and 36 (76.6%) were classified as the SS group. Of the M group patients, 12 patients (15.2%) were classified as MM-pN1, 67 patients (84.8%) were classified as MM-pN2,3. Using another system to subdivide the M group, 55 patients (69.6%) were classified as MMM group, and 24 (30.4%) were classified as MMS group. In the present lymph node metastasis classification system, M-group patients preferentially comprised those with cancer arising from the lower or upper thoracic esophagus (p=0.036), cases with advanced invasion depth (p<0.0001), cases with lymphatic vessel invasion (p<0.0001) and cases with blood vessel invasion (p<0.0001) (Table II).

**Figure 2:**
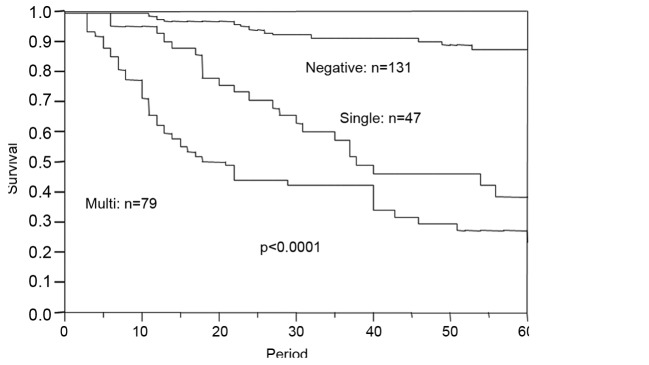
Survival based on the number of metastasis-positive lymphatic stations.

**Figure 3:**
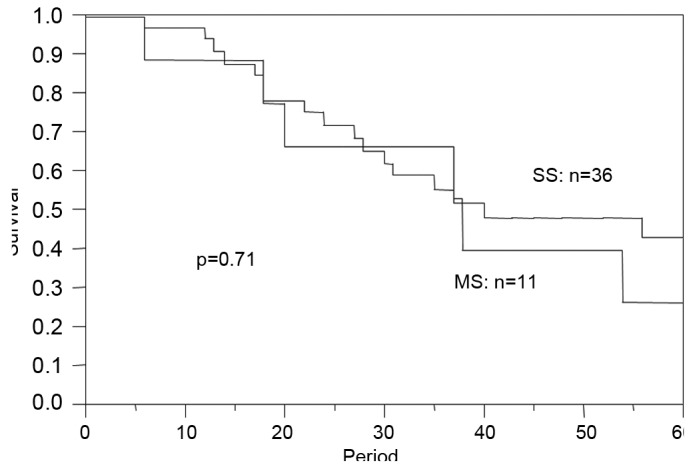
Survival of the patients with single metastasis-positive lymphatic stations. SS, single-station; MS, multi-station.

**Figure 4:**
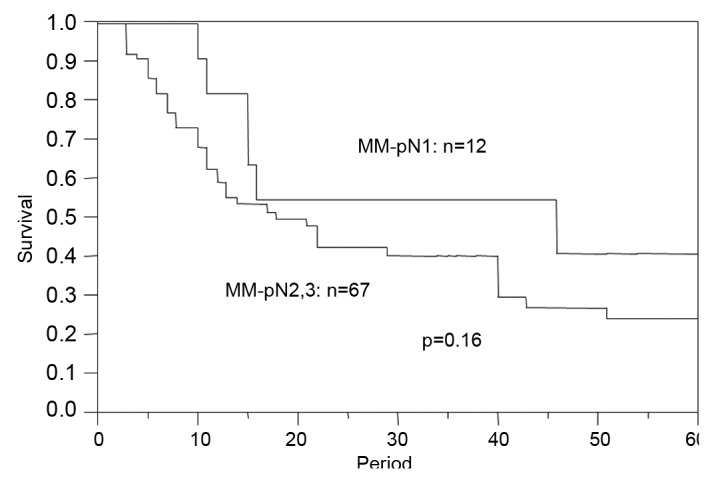
Survival of the patients with multi-metastasis-positive lymphatic stations. MM-pN1, multi-station in pN1; MM-pN2,3, multi-station in pN2 or 3.

**Figure 5:**
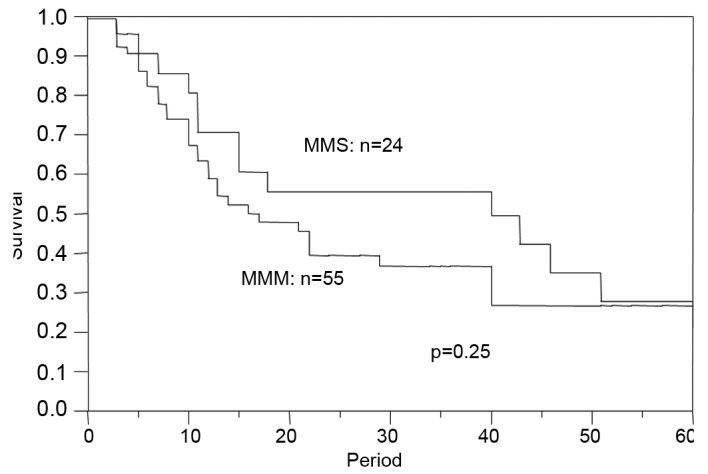
Survival of the patients with multi-metastasis-positive lymph nodes. MMS, multi-station-single-area; MMM, multi-station-multi-area.

Lymph node metastasis classification in the present study revealed a distinct prognostic significance (p<0.0001). For example, multiple-station metastasis was a significant negative prognostic parameter compared with single-station metastasis (p=0.0035) (Fig. 2). However, prognosis was similar when comparing the MS and SS groups (p=0.71) (Fig. 3). In the MS group, the number of positive lymph nodes ranged from 2 to 8 (mean was 3.09). Five cases with more than 3 positive nodes were included in the MS group, 4 cases were classified as pN2 and 1 case was classified as pN3 in the TNM classification. Furthermore, there was no significant difference in prognosis when comparing the MM-pN1 and MM-pN2,3 groups (p=0.16) or when comparing the MMM and MMS groups (p=0.25) (Figs. 4 and 5).

Notably, the association between prognosis and node metastasis classification in this study (p<0.0001) was similar to that between prognosis and lymph node involvement of the TNM and Japanese classification systems in the multivariate analysis including conservative clinicopathological prognostic parameters despite close interaction with each other (Table III).

## Discussion

TNM classification can be used to predict prognosis in patients with esophageal cancer according to cancer stage (3). In the 6th edition of the TNM classification, lymph node involvement is classified as either present or absent. However, the 7th edition of the TNM classification, published in 2010, incorporates the number of lymph nodes with metastatic involvement and may be a more accurate prognostic parameter. This revised system still does not acknowledge the anatomical lymphatic spread, which may limit its overall utility. Thus, the present study utilized the revised TNM system in combination with the Japanese classification, which does assess anatomical lymphatic spread of metastasis. This node classification has prognostic significance, but the lymph node station category can vary with tumor location despite having the same lymph node station (4). Therefore, the present study used a simple modification of this system, in which the pN category of the lymph node station is not determined in detail, but the location of lymph node station is still taken into account.

In this study, patients with multi-station lymph node metastasis preferentially comprised those with cancer arising from the lower or upper thoracic esophagus (p= 0.036), cases with advanced invasion depth (p<0.0001), cases with lymphatic vessel invasion (p<0.0001) and cases with blood vessel invasion (p<0.0001). Lamb *et al* and Kim *et al* reported that multiple sentinel nodes were detected more frequently in patients with lower thoracic esophageal cancer, which may account for the implied finding that lower thoracic esophageal cancers are more prone to metastasize to multiple nodes or stations (5,6). However, there has been no previous report suggesting that upper thoracic esophageal cancers are associated with a higher metastasis-positive station. Concerning the correlation between tumor invasion and lymphatic spread, Feith *et al* reported that deeper tumor invasion was associated with the increased number of metastasis-positive lymph nodes in patients with Barrett's esophageal adenocarcinoma (7). By contrast, the present result, which took into account the number of stations, may be consistent with the previous findings despite the fact that the majority of cases in the present study consisted of squamous cell carcinoma. This indicates that deeper invasive cancer in the lower thoracic esophagus requires much more extended lymph node dissection regardless of the histological type of esophageal cancer.

In the univariate analysis of the prognostic impact, the lymph node metastasis classification system utilized in the present study exhibited a distinct prognostic significance when comparing the S and M groups, and the M group exhibited less favorable prognosis following surgery. Several other lymph node classification systems have been proposed to predict outcomes in patients undergoing surgical management of esophageal cancer. For example, Roder *et al*, Eloubeidi *et al* and Wilson *et al* described the utility of involved lymph node ratios for predicting an unfavorable prognosis (8–10). Roder *et al* and Eloubeidi *et al* set the cut-off values at 20 and 10%, and the increased ratio of metastatic nodes revealed an unfavorable prognosis. However, when Wilson *et al* set the cut-off values at 25 and 50%, there was no difference in prognosis when comparing the two groups. Altorki *et al* suggested that the increased number of dissected lymph nodes in the context of extended lymphadenectomy resulted in a decreased positive node ratio and a more favorable prognosis (11). By contrast, Dhar *et al* reported that the longer diameter of the largest metastatic lymph node was a strong negative prognostic factor, whereas Komori *et al* emphasized that the size of the cancer nest in the lymph node, but not lymph node size, had a prognostic impact (12,13). These previous reports were limited by the fact that they only examined very limited lymph node metastatic mode parameters. However, the present study takes into account the number of positive nodes represented by the TNM classification and the anatomical lymphatic spread represented by the Japanese classification, which likely resulted in a stronger independent prognostic factor, even within multivariate analysis including conservative pathological parameters.

Notably, there was no prognostic difference between the SS and MS groups in this study. This indicates that a favorable prognosis may occur when lymph node metastasis is limited to a single station, even in the context of pN2 or 3 status in the TNM classification. Furthermore, lymph node dissection may be very effective in cases with limited anatomical lymphatic spread (e.g., S group), since all cases employed in the present series were surgically resected with extended curative lymph node dissection. Prognosis was also similar when comparing MM-pN1 cases and MM-pN2,3 cases, which supports inclusion of these two subclassifications within the same M group. The results of the subgroup analysis also indicate that anatomical lymphatic spread and the number of metastasis-positive nodes play an important role in outcome following surgical treatment of esophageal cancer, although Kunisaki *et al* reported that the number of metastatic nodes provides a more accurate estimate of prognosis than the anatomical lymphatic spread (14).

The lymph node metastasis classification system used in the present study was an independent prognostic parameter, even within multivariate analysis including conservative pathological parameters, such as tumor invasion depth and lymphatic or blood vessel invasion. This result suggests that this classification system is an effective alternative to the pN category in the TNM or Japanese classification.

In conclusion, lymph node metastasis classification based on the number of metastasis-positive stations is a useful predictor of prognosis in patients undergoing surgical management of esophageal cancer. This system relies on a simple classification method that combines the Japanese classification based on lymphatic spread and the TNM classification based on the number of positive nodes.

## Figures and Tables

**Table I:** Characteristics of the patients undergoing thoracoscopic surgery for esophageal cancer.

Characteristic	No. of patients
Age (years)	64 (36–84)
Gender	
Male	224
Female	33
Tumor location	
Upper thoracic	32
Middle thoracic	143
Lower thoracic	82
Histology	
Squamous cell carcinoma	228
Adenosquamous carcinoma	5
Adenocarcinoma	9
Basaloid carcinoma	10
Spindle cell carcinoma	1
Neuroendocrine carcinoma	1
Small cell carcinoma	2
Undifferentiated carcinoma	1
Tumor depth (pT)	
*in situ*	5
1	116
2	35
3	99
4	2
Lymph node metastasis (TNM classification)	
0	131
1	55
2	46
3	25
Lymph node metastasis (Japanese classification)	
0	131
1	42
2	46
3	19
4	19
Lymphatic vessel invasion	
Negative	108
Positive	149
Blood vessel invasion	
Negative	179
Positive	78

**Table II:** Correlation between lymphatic spread and clinicopathological factors.

	Lymphatic spread			p-value
	Negative	Single station	Multi-station	
Gender				
Male	116	41	67	0.74
Female	15	6	12	
Age (years)	62.8	65.2	65.4	
Location				
Lower	35	17	30	0.036
Middle	85	21	37	
Upper	11	9	12	
Tumor invasion (pT)				
Superficial (pTis, 1)	95	18	8	<0.0001
Advanced (pT2, 3)	36	29	71	
Lymphatic vessel invasion				
Negative	82	16	10	<0.0001
Positive	49	31	69	
Blood vessel invasion				
Negative	109	34	36	<0.0001
Positive	22	13	43	

**Table III:** Multivariate analysis of prognostic impact with other clinicopathological parameters.

	Risk ratio	95% Confidence interval	p-value
Tumor invasion (pT)	4.412	0.6989–15.36	0.049
Lymphatic vessel invasion	0.6984	0.4915–0.9611	0.027
Blood vessel invasion	1.075	0.8291–1.391	0.58
No. of positive LN stations	1.919	1.359–2.737	<0.0001

LN, lymph node.
